# Growth Performance, Haematobiochemical Profile and Enteric Methane Emission of Goats Supplemented With *Terminalia brownii* Dried Leaves

**DOI:** 10.1002/vms3.71312

**Published:** 2026-11-03

**Authors:** Berihun Kibret, Bimrew Asmare, Likawent Yeheyis, Shigdaf Mekuriaw

**Affiliations:** ^1^ Department of Animal Science College of Agriculture and Natural Resources Mekdela Amba University Tulu Awulia Ethiopia; ^2^ Department of Animal Science College of Agriculture and Environmental Science Bahir Dar University Bahir Dar Ethiopia; ^3^ Amhara Agricultural Research Institute Bahir Dar Ethiopia; ^4^ Unique land use GmbH ILRI Campus Addis Ababa Ethiopia

**Keywords:** average daily gain, digestibility, supplementation, *Terminalia brownii*

## Abstract

**Background:**

Goats are vital for livelihoods and food security in tropical regions such as Ethiopia. However, poor‐quality feed, especially during dry seasons, limits their growth, health and overall productivity.

**Objective:**

To evaluate the effect of supplementing *Terminalia brownii* tree leaves on growth performance, hematobiochemical profile and methane (CH_4_) emission of goats.

**Methods:**

Twenty‐five male goats with an average initial weight of 19.1 ± 1.6 kg were randomly assigned to five dietary treatments, each with five replicates. The treatments included a control group fed 100% concentrate mix (CM) (T_1_), 85% CM + 15% *Terminalia brownii* (T_2_), 70% CM + 30% *Terminalia brownii* (T_3_), 55% CM + 45% *Terminalia brownii* (T_4_) and 40% CM + 60% *Terminalia brownii* (T_5_). All goats were provided with natural pasture hay as a basal diet. After 90 days of feeding, a seven‐day digestibility trial was conducted to assess nutrient digestibility. CH_4_ emission was measured by laser methane detector tool. The data of nutrient content, nutrient intake, digestibility, body weight gain, feed conversion efficiency (FCE), blood profile and CH_4_ emission were recorded. Data were analysed using SAS (version 9.4) software.

**Result:**

The result showed that goats that received the T_5_ treatment consumed significantly more DM (779.24 g) and CP (181.06 g) than other treatment groups. The digestibility of NDF and ADF was not statistically different among the treatments. The average daily weight gain of goats reared in T_5_ (63.4 g and T_4_ (59.0) was higher than T_1_, T_2_ and T_3_. Goats fed T_5_ recorded the highest count of lymphocytes (LYM) (61%) followed by animals fed T_3_ (58.17%) while the least (36.5%) was observed in T_4_ treatment. Besides, goats fed T_4_ diet had the highest neutrophil count (56.9). Goats fed on T_5_ diet had the highest haemoglobin levels as compared to other treatments. The CH_4_ value was lower in T_5_ (34.84 ppm‐m), where goats were fed with 60% *Terminalia brownii* leaves, in comparison to the other treatments.

**Conclusion:**

This indicates that *Terminalia brownii* leaves could potentially act as a viable alternative to concentrate feeds while also reducing methane emissions. This suggests that the T_4_ and T_5_ treatments effectively promoted both growth and feed intake in the goats. In addition, substituting *Terminalia brownii* leaves with concentrate feed had no adverse effect on the blood profile of goats. Future studies should incorporate a comprehensive cost‐benefit analysis to determine whether the potential improvements in goat performance and environmental benefits offset the costs of harvesting and processing *Terminalia brownii* leaves.

## Graphical abstract

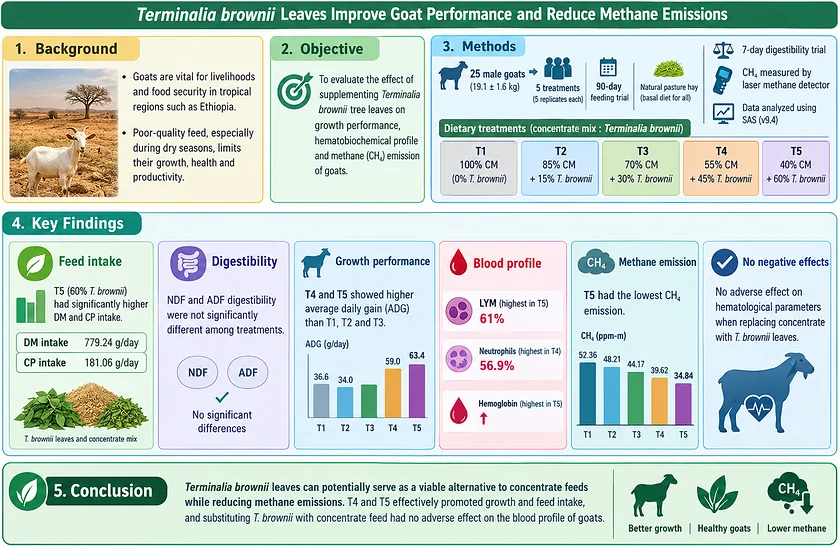

## 1 Introduction

In Ethiopia, low‐quality roughages, such as natural pastures and crop residues, are the primary sources of livestock feed (Gashaw and Defar 2017; Begna and Masho 2024). However, these feed resources are low in nutrients with poor digestibility. Of these limitations, the most prevalent is protein deficit from such feeds, and this intron can be compromised with a straightforward source of nitrogen supplements (Chen et al. 2022). Besides, low‐quality feed contributes substantially to CH_4_ emissions from livestock digestive systems (Vargas et al. 2022). Ruminant animals lose between 2% and 12% of their consumed energy through CH_4_ gas production, negatively impacting their overall productivity (Johnson and Johnson 1995). This energetic loss is particularly relevant in the tropics, where natural pasture and crop residues are the main source of ruminant feeds. Dietary supplementation of poor‐quality diets is known to improve intake by increasing nitrogen supply to rumen microorganisms (Karimi et al. 2021; Morales et al. 2021). This in turn has a positive effect on increasing the microbial population and digestion rate of feed. As the rate of breakdown of digested feed increases, intake is accordingly increased (Cammack et al. 2018; Liu et al. 2019).

Conventional concentrate supplements are accessible at a high price in developing countries (Dey et al. 2022; Melesse et al. 2023). Among these, by product feeds such as oil seed meals, sugarcane bagasse and brans from the edible oil, sugar and flour processing units could help to address the feed challenges in Ethiopia (Begna et al. 2023). However, those concentrated feeds are expensive and not reasonable for smallholder farmers of the tropics. The foliage of trees and shrubs appears to be a sustainable alternative to supply economically affordable supplements for smallholder livestock producers without negatively affecting the health of the animal and environment (Kholif et al. 2022). Browse species have been reported to increase protein supply in roughage feeds and improve digestibility by optimising rumen fermentation (Ebrahim and Negussie 2020; Singh et al. 2023). Besides, improving nutritive values of low‐quality feeds, the inclusion of tree and shrub leaf meal may help reduce CH_4_ gas emission. CH_4_ gas emission from ruminants is a global concern. Subsequently, the browse species is recommended for supplementing low‐quality roughages. Furthermore, haematobiochemical examination is among the methods, which may contribute to the detection of some changes in health status, which may not be apparent during physical examination but which affect the fitness of the animal (Vorobyov et al. 2019). For instance, fodder trees and shrubs have been found to have a good crude protein (CP) content and are used as supplements for ruminant animals in different parts of the world (Sisay et al. 2018; Almaz et al. 2021; Abraham et al. 2023).

One of the browse fodder used as a protein source is *Terminalia brownii*. It is a native tree of tropical Africa. The leaves of the tree are a good source of protein for livestock production. Its leaves are rich in protein, with levels ranging from 12.27 % to 16.94 % (Milkias and Derrebe 2022; Adane et al. 2023). This substantial amount of CP makes valuable feed resources for animal production. In line with this, in the current study area, typically in the lowlands of Ebinat district, Northwest Ethiopia, *Terminalia brownii* tree is widely available and found to be a source of succulent fodder, especially in the dry season when pastures and crop residues deplete in their nutritive values. However, there was no documented information on *Terminalia brownii* leaves for ruminant nutrition and the influence of *Terminalia brownii* diets on the hematobiochemical indices of small ruminants. Therefore, the objective of this experiment was to evaluate the effect of replacing the concentrate mixture with *Terminalia brownii* leaves on the growth performance, blood profile and CH_4_ emission of goats.

## 2 Materials and Methods

### 2.1 Description of the Experimental Site

The experiment was conducted in Bahir Dar University, College of Agriculture and Environmental Science animal farm. The site is situated at an elevation of 1912 m above sea level and is positioned between latitude 11°37'N and longitude 37°28'E. The average daily maximum and minimum temperature was 29°C and 7°C respectively. The average annual rainfall is 1445 mm. The main rainy season is from June to September.

### 2.2 Experimental Animals and Their Management

Twenty‐five male goats with an average age of one year were bought from Ebinat town, South Gondar zone, Ethiopia. Dentition and information from the owners were used to estimate the age of the experimental animals. For identification, the experimental animals were given ear tags. The experimental goats were kept for 15 days to adapt the experimental feeds. All experimental goats received treatments (albendazole and Ivemectin 1%, 1 mL/50 kg single dose) against internal and external parasites. Throughout the trial, the prevalence of illnesses and abnormalities on the animals was regularly monitored. The sample size was determined considering the availability of experimental goats and the practical and resource constraints associated with conducting a controlled feeding experiment.

Randomised complete block design (RCBD) was used for this experiment. Based on their initial body weight, the experimental goats were grouped into five blocks of five goats each. The experimental goats from each block were randomly assigned to each treatment feed. Thus, each treatment was replicated five times, with a total of five goats per treatment. The goats were fed individually, and each goat was considered an experimental unit. The results of nutrient content data of feed ingredients were used to formulate the five experimental feeds that are presented in Table 1. In T_1_, T_2_, T_3_, T_4_ and T_5_, *Terminalia brownii* was used as a replacement for 0%, 15%, 30%, 45% and 60% of the concentrate mixture, wheat bran, nouge seed cake and maize grain, respectively. Soon after the formulation, representative samples were taken from each of the treatment feeds for conducting the complete chemical composition of five treatment feeds.

**TABLE 1:** Allocation of experimental treatments.

Feed ingredients	T_1_	T_2_	T_3_	T_4_	T_5_
Natural pasture hay	Ad libitum	Ad libitum	Ad libitum	Ad libitum	Ad libitum
**Composition of concentrate mix (% of DM)**					
Noug seed cake	21	17	14	12.50	9.75
Wheat bran	60	52	42	30	19.50
Ground maize grain	18	15	13	11.50	9.75
*Terminalia brownii*	0	15	30	45	60
Common salt	1	1	1	1	1
**CP and ME content of concentrate mix**					
CP, % of DM	17.94	17.40	16.90	16.65	16.22
ME, MJ/kg DM	8.88	8.20	7.80	7.22	6.62

*Note*: T_1_ = Hay + 100% CM; T_2_ = Hay + 85% g CM + 15% *Terminalia brownii*; T_3_ = Hay + 70% CM + 30% *Terminalia brownii*; T_4_ = Hay + 55% CM + 45% *Terminalia brownii* and T_5_ = 40% CM + 60% *Terminalia brownii*.

Abbreviations: CP, crude protein; ME, metabolizable energy.

### 2.3 Feeds and Feeding Management

The basal feed used in this study was natural pasture hay (NPH) consisting of grasses and some herbaceous legumes. Leaves of *Terminalia brownii* were collected from the backyard area of Ebinat district in South Gondar zone, Ethiopia. It is located in between 11° and 12° north latitude, and 37° and 38° east longitude. The *Terminalia brownii* leaves were transported to the experimental site and air‐dried under a shed. After drying, the leaves were mixed and chopped by hand to prevent selectively eating only certain parts. Similarly, the pasture hay was also chopped to encourage the goats to consume all parts of the feed. Maize grain was purchased from local market, and nouge seedcake and wheat bran were bought from oil extraction and flour meal factories in Bahir Dar town. The basal diet was offered ad libitum along with free access to drinking water. The proportion of concentrate supplements, that is, maize grain, noug seedcake and wheat bran, were determined based on the comparable the iso‐ nitrogen calculation. Manually chopped *Terminalia brownii* leaves were mixed with the basal diet and provided to the experimental animals.

### 2.4 Dry Matter and Nutrient Intakes

The researchers carefully monitored the experimental goats and daily recorded both the amount of feed offered and refused. The daily feed intake was determined by subtracting the refused feed from the offered amount for each goat. The total dry matter and nutrient intakes were determined by multiplying the total daily feed consumed by the nutrient composition of the diet.

### 2.5 Dry Matter and Nutrient Digestibility

The digestibility of the treatment feed was done after the 90‐day feeding trial for 7 days. Experimental goats were harnessed with faecal collection bags. Faeces were collected every morning; 20% of representative samples were taken and frozen at −20°C and pooled during the collection period for each goat. At the end of the collection period, each sample was mixed and dried at 60°C for 72 h to determine chemical analysis. The apparent digestibility of nutrients was determined as the proportion of the difference between the nutrient consumed and the nutrient in the faeces.

$$\mathrm{Apparent}\;\mathrm{DM}\;\mathrm{digestibility}=\frac{\mathrm{DMI}-\mathrm{Faecal}\;\mathrm{DM}\;\mathrm{output}}{\mathrm{DMI}}\times 100$$

$$\begin{array}{ccc} & & \mathrm{Apparent}\;\mathrm{nutrient}\;\mathrm{digestibility}\\ & & \;=\frac{\mathrm{Nutrient}\;\mathrm{intake}-\mathrm{faecal}\;\mathrm{nutrient}\;\mathrm{output}\;}{\mathrm{nutrient}\;\mathrm{intake}\;}\times 100 \end{array}$$

### 2.6 Body Weight Change

The initial body weight of goats was measured by taking the body weights during the start of the experiment. Their weight was taken every two weeks in the morning before feeding. The final weight was measured at the end of the experiment. The average daily gain (ADG) was determined as the change between the final and initial weights divided by the number of days of feeding. FCE was estimated as a ratio of feed intake to weight gain.

### 2.7 Chemical Composition Analysis of Feeds and Faeces

The representative samples of feed offered and refusals were taken for chemical analysis. The DM, ash and CP contents of feed offered, refusals and faeces were analysed according to methods suggested by AOAC (1990). The dry matter content was determined by drying the samples at 105°C. The nitrogen content of the feed and faeces samples was determined using the Kjeldahl procedure. The CP content was determined by using the calculation N*6.25. Ash was determined by igniting the samples in muffle furnaces at 550°C overnight (AOAC 1990). Neutral detergent fibre (NDF), acid detergent fibre (ADF) and acid detergent lignin (ADL) content was investigated by using Van Soest et al. (1991).

### 2.8 Blood Sample Collection and Analysis

Blood samples were collected from all goats using a blood collection needle. Blood sample was collected in a purple‐top 5 mL vacuum tube containing 10% anticoagulant ethylene diamine tetra‐acetic (EDTA). The blood samples were taken from the jugular vein at the morning before feeding. All the blood samples were taken immediately to the laboratory. The blood haematological tests were read (analysed) by automated haematology analyser. The haematological parameters were packed cell volume (PCV), haemoglobin (Hgb), white blood cells (WBC), differential white blood cells (neutrophil, lymphocyte and monocytes), red blood cells (RBC), mean corpuscular volume (MCV) and mean corpuscular haemoglobin concentration (MCHC).

The blood samples for the serum biochemical analysis were also taken from goats in red‐top vacuum tubes of 5 mL with no anticoagulant that allows for clotting. The blood samples were centrifuged at 3000 rpm with a centrifugation force of 1620 *g* for 15 min. Then after, the serum and plasma samples were transferred to labelled glass vials and stored at a temperature of −20°C pending analysis. An automated biochemical analyser using a commercial kit did the blood biochemical parameters. The separated serum and plasma samples were analysed for biochemical parameters (glucose, cholesterol and total protein (TP)).

### 2.9 Enteric Methane (CH_4_) Emissions Measurement

Enteric CH_4_ emissions were quantified non‐invasively using a hand‑held portable laser methane detector (LMD) employing laser absorption spectroscopy (SA3C32B, Tokyo Gas Engineering Co. Ltd., Tokyo, Japan). All measurements were conducted within a naturally ventilated sheep barn to maintain ambient air exchange, with the goats loosely restrained in a standing position to minimise stress and movement.

To standardise the optical path length and ensure data reproducibility, a rigid measuring rod was affixed to the LMD housing to maintain a constant sampling distance of 1 m between the instrument's receiving lens and the external nares (muzzle) of each goat. The green targeting laser beam was oriented perpendicularly to the exhaled breath plume to capture the maximum path‐integrated methane concentration. For each individual measurement, the LMD recorded continuous CH_4_ concentrations (in ppm‐m) over a 3‑min interval at a sampling frequency of 1 Hz. Extreme or irregular observations associated with temporary disruption of the breath plume or laser alignment were excluded from the calculation of the session mean. The corresponding ambient/background CH_4_ concentration measured 30 s before the goat measurement and was subtracted from the session mean to obtain the background‐corrected CH_4_ concentration:

$${\text{CH}}_{4}\left(\text{corrected}\right)={\text{CH}}_{4}\left(\text{goat}\right)-{\text{CH}}_{4}\left(\text{ambient}\right)$$

where CH_4_ (corrected) is the background‐corrected methane concentration, CH_4_ (goat) is the mean CH_4_ concentration of the stable observations during the goat measurement and CH_4_ (ambient) is the ambient/background methane concentration measured immediately before the goat measurement.

The measurements were conducted across five consecutive days to account for variability. On each day, CH_4_ emissions were recorded at three distinct time points to capture variations linked to rumen fill and feeding dynamics: (i) 09:00 AM (2 h post‑morning feeding), (ii) 1:00 PM (mid‑interval phase) and (iii) 5:00 PM (immediately prior to the evening feeding). This schedule yielded 15 discrete measurement sessions per goat over the experimental period. All data were stored digitally on the device's internal memory and subsequently exported for statistical analysis, where the average CH_4_ emission for each time point and day was calculated per animal (Sorg 2021).

### 2.10 Data Analysis

The data were analysed using the general linear model (GLM) procedures of SAS (version 9.4). Means that showed significant differences among them were separated using the Tukey's Multiple Range test. Differences were considered statistically significant at 5% level of significance. The analysis model was as follows:

$$Y_{\mathrm{ij}}=\mu +T_{i}+B_{j}+e_{\mathrm{ij}}$$

where *Y*_ij_ is the response variable (the observation in j^th^ block and i^th^ treatment), μ is the overall mean, *T*_i_ is a treatment effect, *B*_j_ is a blocking effect and *e*_ij_ is a random error.

## 3 Results

### 3.1 Chemical Composition of Experimental Feeds

The chemical composition of experimental feeds and refusals is shown in Table 2. In the current study, the CP content of *Terminalia brownii* (15.80%) had a higher content as compared to the CP content of the offered NPH (7.64%). Likewise, *Terminalia brownii* showed lower NDF, ADF and ADL (38.63%, 16.96% and 3.35%) contents, respectively, than NPH. In terms of CP content, *Terminal brownii* (15.8% CP) was comparable with wheat bran (16%). However, noug seed cake (31.15%) had a better value of CP as compared to *Terminalia brownii* leaves. Ash content varied throughout the diets, from 5.28% to 14.14% having lower and higher contents of ash in wheat bran and noug seed cake, respectively.

**TABLE 2:** Chemical composition of the experimental feed ingredients and treatments (%).

	DM	OM	Ash	CP	NDF	ADF	ADL
**Feed ingredients**							
NPH	91.59	88.24	11.76	7.64	71.12	49.46	13.75
*TB*	92.63	90.30	7.74	15.80	38.63	16.96	3.35
NSC	92.26	91.26	14.14	31.15	38.10	31.74	10.74
WB	94.15	91.70	5.28	16	49.24	15.56	5.68
GMG	91.9	89.8	8.8	10	41.54	21.87	4.17
**Treatment feeds**							
T1	92.50	90.60	7.40	17.15	55.4	42.60	11.05
T2	92.70	91.23	7.77	17.07	42.50	34.83	7.43
T3	93.40	91.20	8.83	16.60	34.90	25.47	8.43
T4	91.75	90.90	9.10	16.30	40.85	32.25	10.40
T5	93.43	91.23	8.87	16.20	34.43	29.0	10.63

*Note*: T_1_ = Hay + 100% CM; T_2_ = Hay + 85% g CM + 15% *Terminalia brownii*; T_3_ = Hay + 70% CM + 30% *Terminalia brownii*; T_4_ = Hay + 55% CM + 45% *Terminalia brownii* and T_5_ = 40% CM + 60% *Terminalia brownii*.

Abbreviations: ADF = acid detergent fibre; ADL = acid detergent lignin; CP = crude protein; DM = dry matter; GMG = ground maize grain; NDF = neutral detergent fibre; NPH = natural pasture hay; NSC = noug seed cake; OM = organic matter; TB = *Terminalia brownii*; WB = wheat bran.

### 3.2 DM and Nutrient Intake

The daily DM and nutrients intake of experimental goats supplemented with *Terminalia brownii* leaves are shown in Table 3. There was statistical difference for both DM and OM intake among treatments. The inclusion level of *Terminalia brownii* leaves increased, and the DM and OM intakes were increased. On the other hand, the lowest DM (608.58 g/day) and OM (563.56 g/day) intake were investigated in the sole concentrated mixture supplemented group, while the highest were for T_5_ (779.24 and 763.68 g/day) group, respectively. A quadratic regression analysis was performed to evaluate the relationship between the inclusion level of *Terminalia brownii* and DMI (Figure 1). The fitted quadratic equation was DMI = 0.06413x^2^ − 1.20762x + 616.377 (*R* ^2^ = 0.9703), where *x* represents the inclusion level (%). Based on the quadratic response curve, the DMI increased quadratically with increasing levels of *Terminalia brownii* leaves. Correspondingly, NDF, ADF and ADL intakes are also meaningfully different among treatments and increased as the inclusion of *Terminalia brownii* increased. The result showed that supplementation of *Terminalia brownii* increased CP intake, and there was an increasing trend as the level of inclusion increased, the highest being in T_5_ (181.06 g/day). As compared to the groups supplemented with as concentrate mixture, T_4_ and T_5_ treatment groups had the highest intake of ash.

**TABLE 3:** Nutrient intake of goat supplemented with different levels of *Terminalia brownii*.

Intake (g/day)	Treatment					Sig.
	T_1_	T_2_	T_3_	T_4_	T_5_	
DMI	608.58^b^	631.62^b^	627.84^b^	686.42^ab^	779.24^a^	**
OMI	563.56^b^	617.24^b^	616.40^b^	711.78^a^	763.68^a^	***
AshI	48.68^d^	54.22^c^	57.96^c^	67.84^b^	76.44^a^	***
CPI	128.30^b^	135.88^b^	142.22^a^	174.24^a^	181.06^a^	***
NDFI	246.02^c^	265.84^bc^	287^b^	343.20^a^	362.52^a^	***
ADFI	174.22^c^	189.44^c^	227.02^b^	270.94^a^	280.30^a^	***
ADLI	52.82^c^	67.84^b^	69.42^b^	72.70^b^	87.38^a^	***

*Note*: Means in the same row with different superscript differ significantly. T_1_ = Hay + 100% CM; T_2_ = Hay + 85% g CM + 15% *Terminalia brownii;* T_3_ = Hay + 70% CM + 30% *Terminalia brownii*; T_4_ = Hay + 55% CM + 45% *Terminalia brownii*; T_5_ = 40% CM + 60% *Terminalia brownii*.

Abbreviations: ADFI = acid detergent fibre intake; ADLI = acid detergent lignin intake; AshI = ash intake; CPI = crude protein intake; DMI = dry matter intake; NDFI = neutral detergent fibre intake; OMI = organic matter intake.

** = (*p < *0.01).

*** = (*p < *0.001).

**FIGURE 1:**
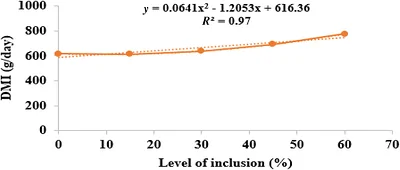
Dry matter intake (DMI, g/day) response of goats to level of inclusion of *Terminalia brownii* dried leaves (%).

### 3.3 Apparent DM and Nutrients Digestibility

The apparent dry matter and nutrient digestibility of the treatment feeds are shown in Table 4. There were notable variations (*p < *0.005) in CP and acid detergent lignin digestibility values among the treatments. The groups treated with T_4_ had a higher CP digestibility (73.96%) than the groups supplemented with T_1_, T_2_, T_3_ and T_5_. There were no statistical variations in OM, NDF and ADF values among the treatments.

**TABLE 4:** Apparent digestibility of DM and nutrients in goats (%).

	Treatments						
Parameters	T_1_	T_2_	T_3_	T_4_	T_5_	SEM	Sig.
DMD	60.97	63.07	64.17	72.15	59.90	4.39	NS
OMD	63.67	65.03	66.21	73.41	60.10	4.82	NS
CPD	52.36^b^	61.82^ab^	64.98^ab^	73.96^a^	65.69^ab^	6.43	***
NDFD	59.09	58.41	60.64	69.32	61.57	8.21	NS
ADFD	60.87	56.03	59.61	67.73	60.33	8.45	NS

*Note*: Means in the same row with different superscripts differ significantly. T_1_ = Hay + 100% CM; T_2_ = Hay + 85% g CM + 15% *Terminalia brownii*; T_3_ = Hay + 70% CM + 30% *Terminalia brownii*; T_4_ = Hay + 55% CM + 45% *Terminalia brownii*; T_5_ = 40% CM + 60% *Terminalia brownii*.

Abbreviations: ADFD = acid detergent fibre digestibility; CPD = crude protein digestibility; DMD = dry matter digestibility; NDFD = neutral detergent fibre digestibility; NS = non‐significant; OMD = Organic matter digestibility; SEM = standard error of mean; SL = Significant level.

*** = (*p < * 0.001);

### 3.4 Body Weight Change and FCE

Table 5 shows the body weight gain and FCE of goats fed *Terminalia brownii* leaves. The initial body weight did not differ statistically (*p* > 0.05) among treatments. As the inclusion of *Terminalia brownii* leaves increased to 60%, there was a noticeable increase of final body weight in the experimental goats. The daily weight gains of goats increased as their substitution level of *Terminalia brownii* increased. The daily weight gain was higher in the goats supplemented with a high level of *Terminalia brownii* leaves. A quadratic regression analysis was determined to evaluate the dose‐response relationship between inclusion level and ADG (Figure 2). The fitted quadratic equation was ADG = 0.00794x^2^ + 0.04781x + 34.651 (*R* ^2^ = 0.913). The quadratic model did not identify the optimum inclusion level for ADG. Within the experimentally evaluated range, ADG increased toward the highest tested inclusion level, with the highest observed ADG (63.4 g/day) at the 60% inclusion level. In addition, the data showed that the FCE of the experimental diets did not differ significantly among treatments. T_1_ and T_2_ exhibited the lowest FCE values among the groups supplemented with *Terminalia brownii*.

**TABLE 5:** Body weight change and FCE of goats supplemented with *Terminalia brownii* leaves.

	Treatment						
Parameters	T_1_	T_2_	T_3_	T_4_	T_5_	SEM	Sig.
Initial BW (kg)	18.96	18.80	19.20	19.24	19.08	0.03	NS
Final BW (kg)	22.24^b^	21.86^b^	22.88^ab^	24.56^a^	24.78^a^	1.43	***
BW change (kg)	3.28^b^	3.06^b^	3.68^ab^	5.32^a^	5.70^a^	1.52	*
ADG (g/day)	36.60^bc^	34.0^c^	41.0^abc^	59.0^ab^	63.40^a^	17.10	*
FCE	0.05	0.05	0.06	0.08	0.07	0.03	NS

*Note*: T_1_ = Hay + 100% CM; T_2_ = Hay + 85% g CM + 15% *Terminalia brownii*; T_3_ = Hay + 70% CM + 30% *Terminalia brownii*; T_4_ = Hay + 55% CM + 45% *Terminalia brownii*; T_5_ = 40% CM + 60% *Terminalia brownii;*

Abbreviations: ADG = average daily gain; BW = body weight; FCE = feed conversion efficiency; NS = non‐significant; SEM = standard error of mean; Sig. = significant level.

*** = (*p* < 0.001);

**FIGURE 2:**
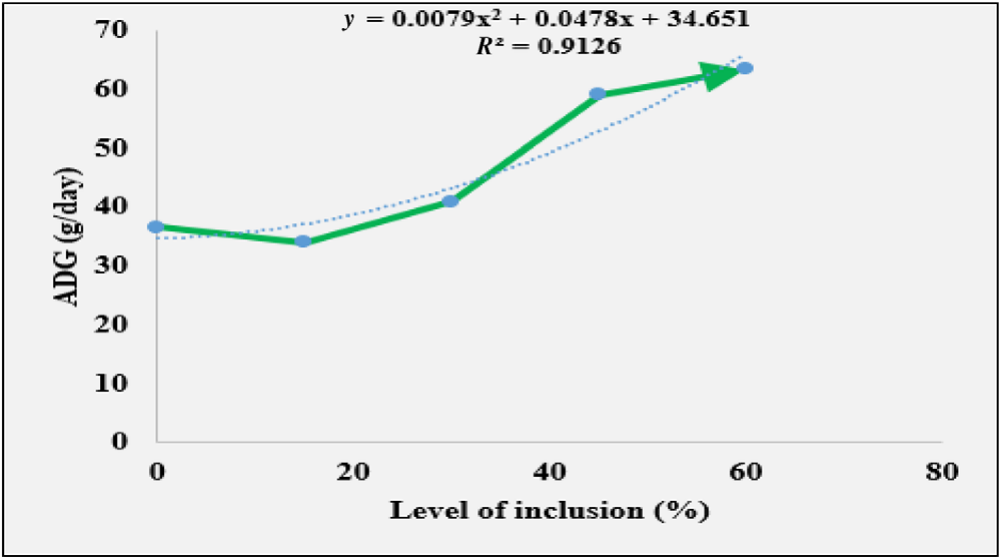
Average daily gain (ADG, g/day) response of goats to level of inclusion of *Terminalia* dried leaves meal (%).

### 3.5 Haematological and Serum Chemistry Parameters of Goats

The haematological profiles of goat fed different levels of *Terminalia brownii* leaves are presented in Table 6. There were no variations (*p* > 0.05) in the haematological parameters except for white blood differentials (lymphocytes, monocytes and neutrophils) and haemoglobin as the inclusion level of *Terminalia brownii* leaves increased in the diet. The differences had been found to be statistically significant (*p < *0.05) for lymphocyte (LYM), monocyte and neutrophil concentration among the treatment groups. Goats fed T_5_ recorded significantly (*p* < 0.05) the highest value of LYM (61%) followed by T3 (58.17%) while the least LYM value (36.5%) was observed in goats fed T_4_ treatment. Besides, the highest neutrophils value (56.9%) had been obtained in T_4_ (with a diet containing 55% concentrate mix with 45% *Terminalia brownii*). Likewise, the results revealed a significant variation in haemoglobin (g/dL) among the treatment groups. Goats fed diet with 60% *Terminalia brownii* (T_5_) leaves meal had the highest haemoglobin concentration (10.74 g/dL) compared with other treatments. Furthermore, there were no significant differences in RBC indices (MCV, MCH and MCHC) observed among the treatment groups. However, the value of MCV (25.95–33.75 (fL)), MCH (8.40–25.10 (pg)) and MCHC (32.75–76.90 (g/dL)) were higher than the laboratory reference values of health goats.

**TABLE 6:** The haematobiochemical profiles of goat fed *Terminalia brownii* leaves.

Parameters	T_1_	T_2_	T_3_	T_4_	T_5_	SEM	Sig.	Ref.
**Haematology**								
WBC (10^3^/µL)	11.21	9.45	10.53	9.64	10.78	2.89	0.96	4−13^a^
Neutrophils (%)	46.10^ab^	39.09^b^	37.84^b^	56.90^a^	36.92^b^	4.08	0.019	30−40^a^
Lymphocytes (%)	46^ab^	57.41^a^	58.10^a^	36.50^b^	61^a^	4.97	0.018	50−70^a^
Monocytes (%)	9.20^a^	3.06^c^	4.65^bc^	5.65^bc^	6.90^ab^	0.75	0.003	0−4^a^
Eosinophils (%)	0.43	0.44	0.33	0.30	1.25	0.29	0.095	1–8^a^
Basophils (%)	0.40	0.45	0.45	0.30	0.40	0.22	0.955	0−1^a^
RBC (10^6^/µL)	11.19	7.52	9.61	4.26	6.89	2.32	0.160	8–18^a^
HGB (g/dL)	9.90^bc^	10.35^a^	8.10^c^	10.40^a^	10.74^a^	0.47	0.014	8–12^a^
MCV (fL)	28.13	31.90	25.95	31.12	33.75	2.17	0.077	16−25^a^
MCH (pg)	9.48	16.65	8.40	25.10	10.26	4.84	0.074	5.2–8^a^
MCHC (g/dL)	33.86	63.40	32.75	76.90	48.28	13.01	0.071	30–36^a^
PCV (%)	24.25	27.50	27.45	28.82	28.87	4.54	0.840	10–30^a^
**Blood chemistry**							
Glucose, mmol/L	1.0	3.0	3.5	4.0	3.0	2.98	0.132	2.4–4^a^
Protein, g/dL	8.0	8.0	7.90	8.65	8.25	0.32	0.266	6.2–7.9^a^
Cholesterol, mmol/L	1.94^a^	1.11^b^	1.55^ab^	1.60^ab^	1.56^ab^	0.15	0.026	1.5–1.9^a^

*Note*: Means with different superscripts (a,b) in the same row are significantly different (*p <*0.05);

Abbreviations: HGB = haemoglobin; MCH = mean corpuscular haemoglobin; MCHC = mean corpuscular haemoglobin concentration; MCV = mean corpuscular value; PCV = packed cell volume; RBC = red blood cell; Ref. = reference value; SEM = standard error of mean; Sig. = significant level; WBC = white blood cell;

^a^ Jackson et al. 2002.

The effects of feeding different inclusion rates of *Terminalia brownii* on serum biochemical profiles of goats are presented in Table 6. Among serum biochemical parameters, cholesterol significantly varied between treatments (*p < *0.05). The control group had significantly (*p < *0.05) higher values of cholesterol (75.0) than other treatment groups. Other indicators, such as TP and glucose levels, demonstrated statistically insignificant differences among treatments.

### 3.6 CH_4_ Emission of Goats Supplemented With *Terminalia brownii* Leaves

The enteric methane emission of goats supplemented with *Terminalia Brownii* dried leaves is presented in Table 7. CH_4_ is a greenhouse gas (GHG) produced and released into the atmosphere in large volumes by ruminants. In this experiment, there was a statistically significant difference in CH_4_ gas emission of goats with the inclusion of *Terminalia browni*i leaf meal. That means goats fed a diet with 60% of *Terminalia brownii* (T_5_) had the lowest (34.83 ppm) CH_4_ gas emissions, while the highest CH_4_ gas emission was observed in goats fed T_1_ (40.28 ppm) supplement diet.

**TABLE 7:** CH_4_ emission of goats supplemented with *Terminalia brownii* leaves (ppm).

	Treatments					
Measurement time	T_1_	T_2_	T_3_	T_4_	T_5_	Sig.
Morning	36.15^abcd^	28.0^d^	33.36^b^	30.53^cd^	30.29^cd^	
Midday	39.13^abcd^	44.02^ab^	32.83^ab^	33.82^bcd^	43.04^ab^	
Evening	45.56^a^	37.91^abc^	40.44^a^	40.86^a^	31.15^cd^	
Time × treatment						**
Overall mean	40.25^a^	36.65^ab^	35.63^bc^	35.07^bc^	34.84^c^	**

*Note*: Means in the same row with different superscript (a, b and c) differ significantly; T_1_(Control) = Hay + 100% CM; T_2_ = Hay + 85% g CM + 15% *Terminalia brownii*; T_3_ = Hay + 70% CM + 30% *Terminalia brownii*; T_4_ = Hay + 55% CM + 45% *Terminalia brownii*; T_5_ = 40% CM + 60% *Terminalia brownii*.

Abbreviation: NS = non‐significant.

**p > 0.05

## 4 Discussion

### 4.1 Chemical Composition of Treatment Feeds

In this experiment, NPH had a low CP content (7.64%). Yet, the CP content of the pasture measured in this research, was higher than the 3.68%–5.9% values investigated by Desta Tekle and Teferi (2017), and it is comparable to the value stated by Abrha et al. (2019). While lower than the values testified by Yeshambel and Bimrew (2018). The NDF, ADF and ADL content of NPH in this study were within the ranges for the majority of tropical fibre feedstuffs. The ADF (49.46%) values of NPH were comparable with 50.70% investigated by Desta et al. (2023). The ADL value obtained from this experiment was less than that of the findings published by (Abrha et al. 2019) but lower than the values reported by Mamo et al. (2023), and Yeshambel and Bimrew (2018). Differences in soil type, altitude and harvesting stage may be the cause of the variation in fibre content between research investigations (Tamrat et al. 2020; Gobena et al. 2022). The fibre content of the basal diet in the current study was within the range of most tropical forages (Lemus 2009). The above result demonstrate that the basal diet is poor in quality for optimum rumen condition and supplementation of this NPH with alternative protein source was recommended.

The CP content observed in *Terminalia brownii* (15.8%) was similar to 14.67% in *Ziziphus* leaves reported by Abrha et al. (2019), but higher than the 12.69% values in *Ficus thonningii* reported by Yeshambel and Bimrew (2018). This difference might be due to season, type of species and age of the plants from where the samples were collected. The NDF, ADF and ADL contents of *Terminalia brownii* found in the current study were lower than the fibre content of browse leaves studied by Misrak et al. (2023), who showed that NDF varied between 43.37% in *Olea africana* and 69.9% in *Cordia africana*, while ADF ranged from 28.56% in *Terminalia brownii* to 53.9% in *Cordia Africana*. Also, higher value of NDF, ADF and ADL of *Terminalia schimperiana* (50.46%, 23.81% and 15.5%) was investigated by Desalegn et al. (2016), respectively in Chilga district in northwestern Ethiopia.

### 4.2 DM and Nutrient Intakes

As the level of *Terminalia brownii* supplementation increased, the total amount of DM intake also increased (see figure 3). This might be the result of supplementing a better rumen environment, which raised microbial protein production and improved DM and nutrient intakes. The results also indicated that improving the total intake in the treatments improves the intake of all the nutrients. The total dry matter and organic matter intakes of supplemented goats in this study ranged from 608.58 to 779.24 and 563.56 to 763.68 g DM/day/head, respectively. The current findings were higher than the findings of Sisay et al. (2022), who reported a range of 556–638 g/ day/ head for DM intakes of Arsi Bale goats supplemented with different browse species. The lowest dry matter intake (593.34 g DM/day) of Arsi Bale goats fed *Chloris gayana* hay supplemented with *Acacia tortilis* browse species was also observed by Sisay et al. (2022). The total DM intake observed in this study was lower than the report of Tekliye et al. (2018). In line with the current study, Balehegn et al. (2014) also investigated that DM and nutrient intake were improved when *Ficus thonningii* leaves were substituted by concentrate supplements in highland goats.

**FIGURE 4:**
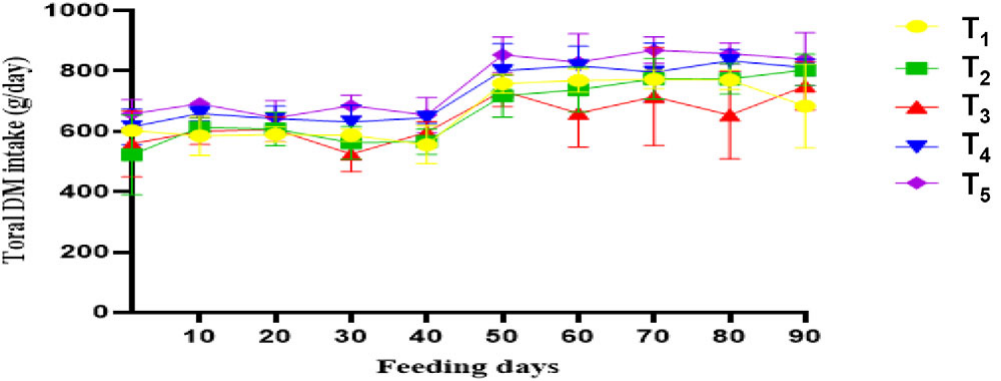
Trends of total dry matter intake of local goat fed natural pasture hay supplemented with graded levels of *Terminalia brownii* leaves. T_1_ = natural pasture hay + 100% CM; T_2_ = natural pasture hay + 85% g CM + 15% *Terminalia brownii*; T_3_ = natural pasture hay + 70% CM + 30% *Terminalia brownii*; T_4_ = natural pasture hay + 55% CM + 45% *Terminalia brownii* and T_5_ = natural pasture hay + 40% CM + 60% *Terminalia brownii*.

The highest DM and OM intake observed in goats fed with *Terminalia brownii* dried leaves might be attributed to the low NDF and ADF values of *Terminalia brownii* leaves. It is commonly acknowledged that the NDF and ADF contents have the greatest impact on fodder intake and degradation. Research has shown that NDF and ADF have a negative link with dry matter intake and digestibility (Mokoboki et al. 2019; Chebli et al. 2021). The present study recorded higher CP intake than those reported by Worku et al. (2015)(54.23–111.98 g/day/ head) in sheep supplemented with *Sesbania sesban* leaves. Ali et al. (2019) also reported an increase in CP intake as the level of sun‐dried *Ziziphus spina‐christi* leaf substitution for NPH increased in Bati goats in Ethiopia, which agrees with the present finding. The higher intake of NDF and ADF in the inclusion group could mostly be a contribution to the fibre content of the supplement.

### 4.3 Apparent Dry Matter and Nutrient Digestibility

The results showed significant differences in CP and ADL digestibility among the treatment groups. Specifically, the group supplemented with T_4_ revealed higher CP digestibility compared to the other treatment groups (T_1_, T_2_, T_3_ and T_5_). However, there were no significant differences in the apparent digestibility of DM, OM, NDF and ADF among the treatments.

The above result aligns with the findings of several previous studies on the effect of browse species supplements on nutrient digestibility in ruminant animals. Dida et al. (2019) found that goats supplemented with 300 g of *Azyhatadirachta indica* leaves had higher nutrient intake and digestibility compared to those supplemented with 300 g of concentrate mix (wheat bran and noug seed cake). Abrha et al. (2019) observed that the apparent digestibility of DM and OM was better for sheep supplemented with *Ziziphus spina‐christi* (400 g) leaves meal, indicating improved nutrient intake. The above result is also in line with the report of McDonald et al. (2021), who noted that the addition of dietary protein in the supplement has increased protein availability to rumen microorganisms to speed up the digestion process. However, some studies have reported contrasting results. Yeshambel and Bimrew (2018) presented that supplementation with higher levels of *Ficus thonningii* leaves meaningfully reduced the digestibility of nutrients. Tasse and Kurniawan (2021) also found that incorporating 5%–10% *Bauhinia purpurea* leaves meal in concentrate mix had no effect on daily DM intake but decreased dry matter digestibility. The discrepancy in these findings could be attributed to plant species, animal breed and mature stage of treatment feeds.

### 4.4 Apparent Dry Matter and Nutrient Digestibility

The highest live body weight of goats supplemented with the *Terminalia brownii* in the current experiment could be due to the higher OM and CP intake of *Terminalia brownii* supplement (see Figure 3). Similarly, Araya et al. (2003) observed higher daily weight gain in goats supplemented with *Acacia tortilis* pods, up to 75% level of inclusion. In line with the present study, Tekliye et al. (2018) found higher average daily body weight gain in Farta sheep supplemented with different levels of *Sesbania sesban* leaves. The ADG of supplemented goats obtained in the present study is lower than the finding of Hagos et al. (2015) in Tigray highland sheep supplemented with *Ziziphus spina‐christi* (90.47 g/day/head) leaves. The ADG measured in the current study was lower than the value reported by Desta et al. (2017) in a Tigray highland ram fed on mixed grass hay ad libitum supplemented with dried mulberry (*Morus indica* L.) leaves. Dida et al. (2023) compared the ADG of sheep fed with different browse diets and reported higher gains in sheep supplemented with a concentrate mixture than in those fed with *Azadirachta indica*, *Stylosanthes fruticosa* and *Dolichos lablab* leaves.

**FIGURE 3:**
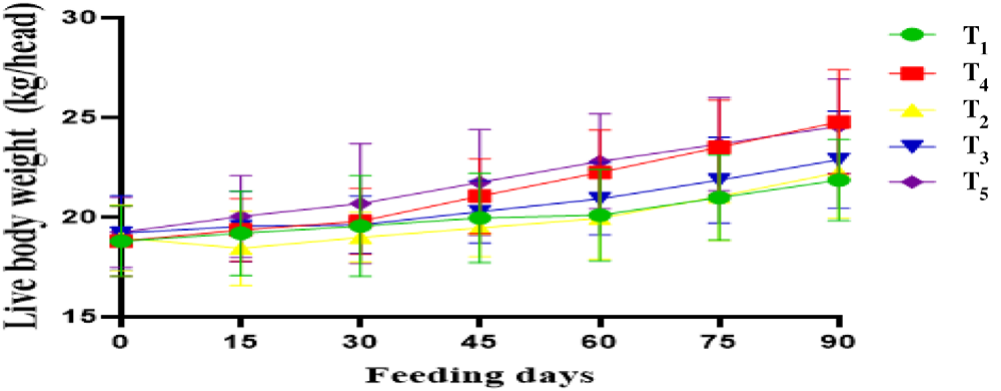
Trends of live body weight of local goat fed natural pasture hay supplemented with graded levels of *Terminalia brownii* leaves. T_1_ = natural pasture hay + 100% CM; T_2_ = natural pasture hay + 85% g CM + 15% *Terminalia brownii*; T_3_ = natural pasture hay + 70% CM + 30% *Terminalia brownii*; T_4_ = natural pasture hay + 55% CM + 45% *Terminalia brownii*; T_5_ = natural pasture hay + 40% CM + 60% *Terminalia brownii*.

Different authors discuss the results of various studies on feed conversion efficiency in small ruminants based on different dietary treatments. The current experiment was in line with Desta et al. (2017), who found no significant difference in FCE among treatments using dried mulberry leaf meal supplementation for sheep. Dida et al. (2019) also showed that FCE was not significantly different among treatments for goats fed with different combinations of *Cajanus cajan* and *Azadirachta indica* supplements.

### 4.5 The Haematobiochemical Profiles of Goat

Blood profiles show an important foundation for evaluating the health condition of animals (Stockham and Scott 2024). The primary functions of white blood cells include producing and distributing antibodies for the immune response as well as protecting the body from foreign organisms (Maddur et al. 2020). According to Jackson et al. (2002), the typical range of WBC for a clinically healthy goat is 4.0–13.0 × 10^9^/L, which is comparable to the values found in this study (9.45–11.21 × 10^9^/L). A high WBC count allows for the production of antibodies and, consequently, a high degree of disease resistance, which is beneficial for adaptability to disease‐prevalent situations (Essien and Udoh 2021). A low WBC count exposed animals to a high risk of infection. When compared to the control group, the lymphocyte count was significantly greater in the 60% *Terminalia brownii* inclusion group. This result was consistent with the findings of Paswan et al. (2016), who demonstrated that the lymphocyte count increased as the amount of *A. nilotica* pod meal in the goat diet increased.

According to Farschtschi et al. (2022), differing lymphocyte values may indicate varied immune status levels in farm animals, and suggests that low lymphocyte numbers may indicate either a weak immune system or a high neutrophil level in an active infection. Lymphocytes play a key role in developing acquired immunity, while neutrophils effectively trap and kill invading pathogens. However, the lymphocyte and neutrophil counts obtained in this study are within the physiological ranges of 47%–82% and 17%–52%, respectively, for apparently healthy goats (Jackson et al. 2002), implying that inclusion of the implying that inclusion of the *Terminalia brownii* leaf parts were not harmful to the goats.

In this experiment, haemoglobin concentration (8.1–10.74) differed significantly among treatments but the values were normal for healthy goats (8.0–12.0 g/dl) as stated by Radostits et al. (2006). This is indicating the absence of anaemia caused by iron deficiency and improper utilisation of iron for the formation of haemoglobin. RBC indices are metrics that are crucial for diagnosing the underlying cause of anaemia (Garcia‐casal et al. 2023). MCV, MCH and MCHC are considered RBC indices that give details about average cell size, haemoglobin content and haemoglobin concentration, respectively (Buttarello 2016). The MCV values of goats 25.95–33.75 (fL) obtained in this study were higher than the reference range by Jackson et al. (2002) for goats 16–25 (fL) of apparently healthy goats. The MCV value obtained in this study implies that the animal is exposed to macrocytic anaemia due to vitamin B12 or folate deficiency (Mohammed et al. 2016). Contrary to expectations, results pertaining to MCH ranged from 8.40–25.10 (pg) in this study were higher than the laboratory reference for haematological parameters of healthy goats of 5.2–8 (pg) (Jackson et al. 2002). An elevated MCH can signify the existence of reticulocytes or haemolysis, whereas a lower MCH is thought to correspond to a severe iron deficiency (Buttarello 2016). Similarly, MCHC has been suggested to be the most accurate of the RBC indices, and it might rise when there is an instance of haemolysis or fall in the case of reticulocytosis and iron deficient anaemia (Buttarello 2016). MCHC values recorded in this study ranged from 32.75–76.90 (g/dL) was higher than the laboratory reference value (30–36) for goats (Jackson et al. 2002).

Serum biochemical parameters are also indicators of the health status of animals. The current study shows no significant effect of diet on TP across all the dietary treatments, however, numerically, goats fed on a diet containing *Terminalia brownii* leaves had higher serum total protein and more comparable to control diet‐fed goats. The concentration of TP found in the present study was in agreement with the values reported by Kholif et al. (2016), who reported that feeding of *Moringa* leaves did not affect serum protein. The concentration of glucose level found in the present study was in agreement with the values reported by Chelopo and Marume (2024). The cholesterol concentration was significantly lower in *Terminalia brownii* leaves fed group as compared to the control groups. Similar to the present findings, Kholif et al. (2016) recorded lower serum cholesterol concentrations in goats fed Moringa leaves in their diets.

### 4.6 CH_4_ Emission of Goats Supplemented With *Terminalia brownii* Leaves

This research explores the replacement of concentrate feed with *Terminalia brownii* leaves to assess its impact on CH_4_ production in goats. The key finding that CH_4_ emissions were statistically different between goats fed the *Terminalia brownii* leaves treatment and those fed a concentrate mix is an important result, as it suggests that *Terminalia brownii* leaves might serve as an effective substitute for concentrate feeds without increasing CH_4_ emissions. To interpret and discuss our findings, it is helpful to compare them with similar studies that have investigated the effects of plant‐based substitutes, fibre sources, and different feeding strategies on methane emissions in ruminants. High‐starch concentrates tend to lead to increased methane emissions because they are rapidly fermented in the rumen, leading to higher hydrogen production, which methanogens utilise to produce methane (Culbertson et al. 2025). On the other hand, browse leaves tend to ferment more slowly and produce less hydrogen, often leading to lower methane emissions (Bonizzi et al. 2025). However, depending on the specific plants used, the presence of secondary metabolites like tannins can either promote or inhibit methane production by affecting the microbial population in the rumen.

The present study revealed a statistically significant (*p < *0.05) reduction in enteric CH_4_ emissions in goats fed *Terminalia brownii* leaves compared with those receiving a conventional concentrate mix. While diets rich in fibrous substrates are generally expected to lower methane output due to delayed fermentation kinetics and reduced hydrogen availability, the observed mitigation in the *Terminalia brownii*‑treated groups suggests that bioactive phytochemicals, most notably condensed tannins or related secondary metabolites, may exert an additional suppressive effect on ruminal methanogenesis beyond that attributable to fibre content alone. Bayssa et al. (2016) reported approximately 6.2% condensed tannins in *Terminalia brownii* leaves, suggesting that the species has the potential to supply tannins at biologically relevant concentrations. This is particularly relevant because tannin‐containing browse species may modify ruminal fermentation by altering the activity and composition of rumen microorganisms, thereby potentially reducing methanogens. This is particularly noteworthy because it indicates that substituting costly concentrate feeds with *Terminalia brownii* leaf meal does not incur an environmental penalty in terms of methane production. From a practical standpoint, this finding carries substantial implications for sustainable small ruminant production, especially in resource‑constrained regions where commercial concentrates are prohibitively expensive or inconsistently available. The strategic deployment of *Terminalia brownii* leaves as a locally accessible feed resource could therefore support environmental stewardship in low‑input livestock systems.

Several studies have investigated the effects of various forage species, particularly browse plants, on CH_4_ emissions. Consistent with our findings on identifying treatment differences using LMD methods, Orzuna‐Orzuna et al. (2021), found that the supplementation of tannin‐rich plants reduced CH_4_ emissions in ruminants. However, the effect varied depending on the type of tannin, the amount included in the diet, and the animal's rumen microbiome. This supports the idea that *Terminalia brownii*, if it contains sufficient levels of bioactive compounds, could modulate CH_4_ production. Leitanthem et al. (2022) showed that *Moringa oleifera* leaves were used as a replacement for concentrated mixtures in goat diets, there was a reduction in CH_4_ emissions, suggesting that browse species could be a viable alternative without increasing CH_4_ output.

## 5 Conclusion

The present finding demonstrate that dietary inclusion of *Terminalia brownii* leaves influenced growth performance, selected haematological parameters and enteric methane emissions in goats. Increasing the inclusion level of *Terminalia brownii* leaves resulted in a progressive improvement in ADG, with goats fed the highest inclusion level (60%) showing the highest weight gain. The inclusion of *Terminalia brownii* leaves also significantly affected selected haematological parameters, particularly lymphocyte and neutrophil counts. Goats fed the 60% of *Terminalia brownii* leaves had the highest lymphocyte count (61%) and haemoglobin concentration (10.74 g/dL), suggesting a favourable influence on some aspects of the haematological profile. Furthermore, CH_4_ emissions differed significantly among treatments, with goats fed 60% *Terminalia brownii* leaves producing the lowest CH_4_ emission (34.83 ppm), compared with the highest value observed in the control treatment (40.28 ppm). Generally, the findings suggest that *Terminalia brownii* leaves have a potential as an alternative feed resource for goats, as their inclusion was associated with improved growth performance and reduced CH_4_ emissions. We therefore recommend strategic incorporation of *Terminalia brownii* leaves into goat rations as a promising climate‐smart feeding strategy. Moreover, future studies should incorporate a comprehensive cost‐benefit analysis to determine whether the potential improvements in goat performance and environmental benefits offset the costs of harvesting and processing of *Terminalia brownii* leaves.

## Author Contributions

**Berihun Kibret**: conceptualization, investigation, writing – original draft, methodology, validation, visualization, writing – review and editing, software, formal analysis, data curation. **Bimrew Asmare**: conceptualization, investigation, writing – review and editing, visualization, validation, methodology, data curation, supervision. **Likawent Yeheyis**: conceptualization, investigation, writing – review and editing, visualization, validation, methodology, supervision. **Shigdaf Mekuriaw**: conceptualization, investigation, methodology, validation, visualization, writing – review and editing, software, data curation, supervision.

## Funding

The authors received funding from the Ethiopian Ministry of Education for this work.

## Ethics Statement

Written informed consent was obtained from the animal owners before the animals were enrolled in the study and subjected to the experimental procedures. All experimental procedures involving animals were conducted in accordance with internationally recognized principles for the care and use of animals in research. The study protocol was reviewed and approved by the Animal Care and Use Committee of Bahir Dar University (Approval No. 14/011/2025). Throughout the experimental period, the animals were housed under appropriate husbandry conditions, monitored regularly for health and welfare, and provided with adequate feed and water. All reasonable efforts were made to minimise discomfort, stress, pain and suffering. Humane handling practices were followed throughout the study, and animal welfare was prioritized at all stages of the experiment.

## Conflicts of Interest

The authors declare no conflicts of interest.

## Data Availability

Data are available with the corresponding author upon reasonable request.
